# Loss of p16 expression is a sensitive marker of *CDKN2A* homozygous deletion in malignant meningiomas

**DOI:** 10.1007/s00401-023-02544-6

**Published:** 2023-02-01

**Authors:** Vivian Tang, Rufei Lu, Kanish Mirchia, Jessica Van Ziffle, Patrick Devine, Julieann Lee, Joanna J. Phillips, Arie Perry, David R. Raleigh, Calixto-Hope G. Lucas, David A. Solomon

**Affiliations:** 1 Department of Pathology, University of California San Francisco, 513 Parnassus Ave, Health Sciences West 451, San Francisco, CA 94143 USA; 2 Clinical Cancer Genomics Laboratory, University of California San Francisco, San Francisco, CA USA; 3 Department of Neurological Surgery, University of California San Francisco, San Francisco, CA USA; 4 Department of Radiation Oncology, University of California San Francisco, San Francisco, CA USA; 5 Present Address: Department of Pathology, Johns Hopkins University School of Medicine, 1800 Orleans St, Sheikh Zayed Tower Room 2101, Baltimore, MD 21287 USA

While meningiomas are generally benign, slow-growing tumors with excellent post-surgical outcomes, a significant subset follows a more aggressive clinical course resistant to existing treatment modalities. Recent genomic profiling studies have led to refined classification schemes that better predict patient outcomes compared to conventional morphologic assessment alone [1, 2, 8]. We now recognize that patients with meningiomas harboring *CDKN2A* homozygous deletion or *TERT* promoter mutation regardless of histologic grade have poor clinical outcomes similar to patients with histologically diagnosed anaplastic (CNS WHO grade 3) meningiomas [10]. As such, the revised definition of anaplastic meningioma in the 2021 WHO Classification of Central Nervous System Tumors now includes any meningioma that harbors *TERT* promoter mutation or *CDKN2A* homozygous deletion [6]. However, there are no uniform recommendations on a standardized molecular workup for meningioma, and the cost of ancillary sequencing remains prohibitive at most institutions. Prior studies have examined p16 expression in meningioma but not in the context of corresponding *CDKN2A* gene status [3, 5]. An unresolved question is whether loss of expression of p16^INK4a^ (the protein product of the *CDKN2A* gene which functions as a negative regulator of cyclin-dependent kinase activity—hereafter p16) by immunohistochemistry can identify likely *CDKN2A*-inactivated meningiomas to be selectively confirmed by follow-up molecular testing.

To explore this, we assembled a cohort of 39 higher grade meningiomas with *CDKN2A* gene status determined by targeted next-generation DNA sequencing (Fig. 1, Supplementary Table 1 [Online Resource 1]). The cohort was composed of 21 males and 18 females, with a mean age at surgery of 57 years (range 16 to 89 years). Tumors were located in the convexity (19), skull base (10), falx/parasagittal (6), spinal cord (3), and metastatic to pelvic bones (1). 24 were initial primary tumors, 14 were recurrent tumors, and 1 was a metastasis. By contemporary morphologic grading criteria, all were histologically higher-grade, either atypical (grade 2, *n* = 27, 69%) or anaplastic (grade 3, *n* = 12, 31%). Targeted capture-based next-generation DNA sequencing was performed using the UCSF500 NGS panel as previously described [4]. Genome-wide copy number and zygosity analysis was performed by CNVkit and visualized using NxClinical (Biodiscovery). Manual review of chromosome 9 and the *CDKN2A* locus was performed in all tumors. An integrated molecular grade based on *TERT* promoter and *CDKN2A* status was assigned for each tumor per the 2021 WHO Classification. Immunohistochemistry for p16 was performed on whole formalin-fixed paraffin-embedded sections for all 39 tumors as described in Methods.

**Fig. 1:**
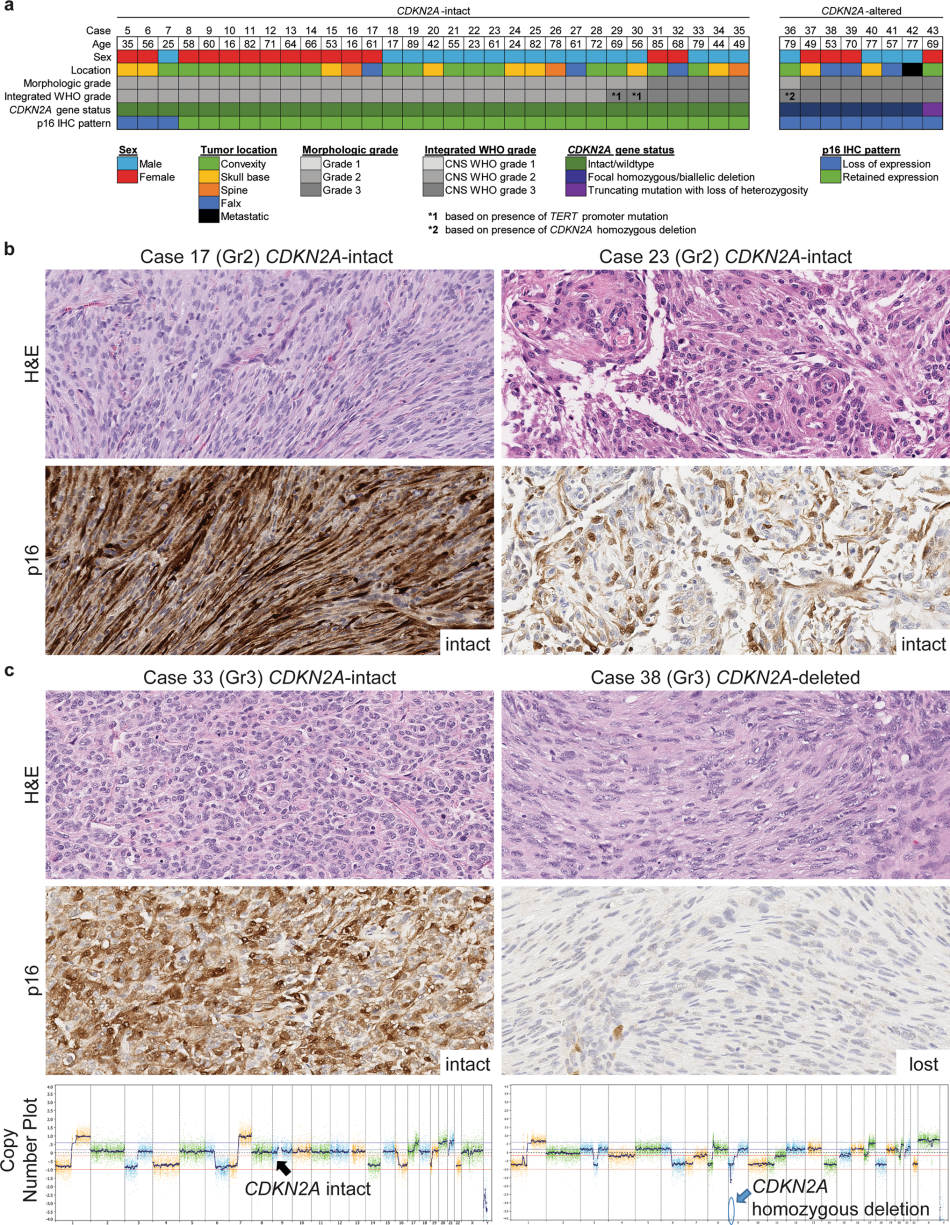
*CDKN2A* gene status and p16 expression in higher-grade meningiomas. **a** Oncoprint table summarizing the clinical characteristics, morphologic grade, integrated WHO grade, *CDKN2A* gene status by targeted next-generation sequencing, and p16 IHC results across 39 atypical (grade 2) or anaplastic (grade 3) meningiomas. **b** In *CDKN2A*-intact tumors, robust p16 expression was observed in a substantial proportion of tumor cells. **c** The CNS WHO grade 3 meningioma from patient 38 demonstrated loss of p16 expression due to *CDKN2A* homozygous/biallelic deletion, while the CNS WHO grade 3 meningioma from patient 33 demonstrated intact p16 expression with intact *CDKN2A* alleles

By targeted DNA sequencing, seven tumors demonstrated focal *CDKN2A* homozygous/biallelic deletion. These seven meningiomas harboring *CDKN2A* homozygous deletion demonstrated complete absence of p16 expression by immunohistochemistry, with intact staining in non-neoplastic endothelial and inflammatory cells (Fig. 1, Supplementary Fig. 1 [Online Resource 2]). An additional meningioma (case #43) harbored an inactivating truncating mutation in *CDKN2A* (p.A60fs) with loss of the remaining wildtype allele which also demonstrated complete absence of p16 expression (Supplementary Fig. 1 [Online Resource 2]). Three meningiomas (cases #5, #6, and #7, all morphologically grade 2) with no identifiable *CDKN2A* alteration had absence of p16 immunoreactivity. The remaining 28 meningiomas with no identifiable *CDKN2A* alteration demonstrated intact p16 expression with immunoreactivity present in a substantial proportion of tumor cells (Fig. 1). These findings translated to an overall sensitivity of 100% (8/8) for p16 loss by immunohistochemistry in detecting *CDKN2A* biallelic inactivation among higher grade meningiomas, a specificity of 90% (28/31), a negative predictive value of 100% (28/28), and a positive predictive value of 73% (8/11).

We also performed p16 immunohistochemistry on 14 CNS WHO grade 1 meningiomas that were all confirmed by DNA sequencing to have intact/wildtype *CDKN2A* alleles (Supplementary Table 2 [Online Resource 1]). The majority of these CNS WHO grade 1 meningiomas (9/14, 64%) demonstrated minimal to absent p16 immunoreactivity (Supplementary Fig. 2 [Online Resource 2]), indicating a poor correlation between p16 expression and *CDKN2A* gene status in benign CNS WHO grade 1 meningiomas which we speculate is due to the low proliferation rate and cell cycle activity. As a result, we do not foresee that p16 immunohistochemistry is an effective screening tool for *CDKN2A* inactivation in CNS WHO grade 1 meningiomas and only recommend p16 immunohistochemical evaluation in higher grade meningiomas.

For morphologically grade 2 meningiomas, identification of an underlying *CDKN2A* homozygous deletion would now lead to an integrated CNS WHO grade 3 designation that has treatment implications including clinical trial eligibility. Among 27 morphologically grade 2 meningiomas in our cohort, four demonstrated loss of p16 expression. Of these four, one was confirmed to harbor *CDKN2A* homozygous deletion and was subsequently increased to CNS WHO grade 3, while the other three had intact/wildtype *CDKN2A* alleles. The remaining 23 histologically grade 2 tumors showed retained p16 expression and intact/wildtype *CDKN2A* alleles. In a resource-limited setting, screening morphologically grade 2 meningiomas with p16 immunohistochemistry can help identify a subset of cases to submit for additional *CDKN2A* assessment and possible change in grade.

For morphologic grade 3 tumors, *CDKN2A* homozygous deletion does not affect grading but may have treatment implications. *CDKN2A* encodes a cell cycle regulatory protein, and ongoing clinical trials are examining the role of CDK4/6 inhibition in meningioma [12]. Among 12 histologically grade 3 tumors, seven demonstrated loss of p16 expression, of which six harbored *CDKN2A* homozygous deletion and one harbored a truncating mutation in *CDKN2A* (p.A60fs). Notably, *CDKN2A* truncating mutations are not currently included in the molecular criteria for CNS WHO grade 3 designation but are likely functionally equivalent to homozygous deletion. Thus, morphologically grade 3 meningiomas demonstrating loss of p16 expression may be considered for further investigation as part of ongoing trials using cell cycle inhibitors such as ribociclib (NCT02933736) and abemaciclib (NCT02523014, NCT03220646). Additionally, cases with intratumoral heterogeneity may only harbor biallelic inactivation of *CDKN2A* in a subset of tumor cells [7, 9, 11]. In such cases, p16 immunohistochemistry may help identify the region of tumor with *CDKN2A* inactivation that should be targeted for microdissection and ancillary confirmatory studies.

In summary, we demonstrate meningiomas harboring *CDKN2A* homozygous deletion or truncating mutation exhibit loss of p16 expression by immunohistochemistry. As such, these results suggest that p16 immunohistochemistry can act as a cost- and time-effective prospective screen of *CDKN2A* gene status in the context of histologically higher grade meningiomas, with loss of p16 expression prompting additional molecular testing to confirm *CDKN2A* mutation or deletion. Future studies are required to define the biologic nature of the subset of meningiomas with loss of p16 expression by immunohistochemistry but an absence of identifiable *CDKN2A* gene alterations.

## Methods for p16 immunohistochemistry

Immunohistochemistry for p16^INK4a^ was performed on whole formalin-fixed paraffin-embedded sections in a Leica Bond autostainer using the mouse monoclonal antibody clone E6H4 from Roche MTM Laboratories following ER1 antigen retrieval. The primary antibody was applied for 30 min at room temperature undiluted at ready-to-use concentration as supplied by the manufacturer. Diaminobenzidine was used as the chromogen, followed by hematoxylin counterstain.

## Supplementary Information

Below is the link to the electronic supplementary material.

Supplementary file1 (PDF 10668 kb)

Supplementary file2 (XLSX 14 kb)

## Data Availability

Digitally scanned image files of representative H&E and p16 immunostained sections are available at the following link: https://figshare.com/projects/Meningiomas_with_and_without_CDKN2A_homozygous_deletion/155759. Detailed clinicopathologic annotations are available in the electronic supplementary material. Raw sequencing data files are available upon request.
