# Supplementary material for: Loss of p16 expression is a sensitive marker of CDKN2A homozygous deletion in malignant meningiomas
Source: Acta Neuropathol. 2023 Feb 1;145(4):497–500. doi: 10.1007/s00401-023-02544-6 (PMC10020299; doi:10.1007/s00401-023-02544-6)
Supplement: Supplementary file 1 — Supplementary file1 (PDF 10668 kb) [file 401_2023_2544_MOESM1_ESM.pdf]

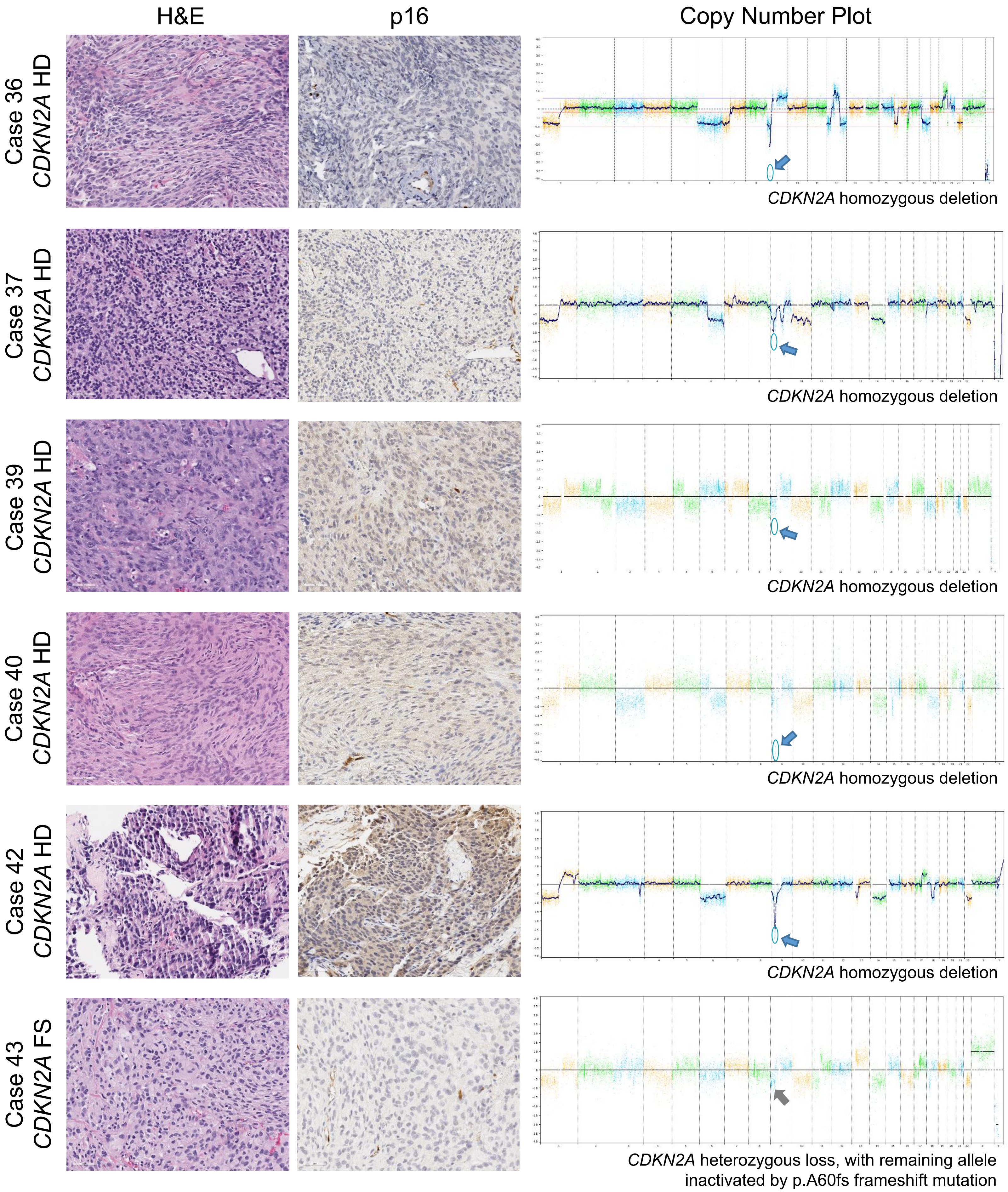

Supplementary Figure 1. Loss of p16 expression in *CDKN2A*-altered meningiomas. Homozygous deletion (HD), frameshift mutation (FS)

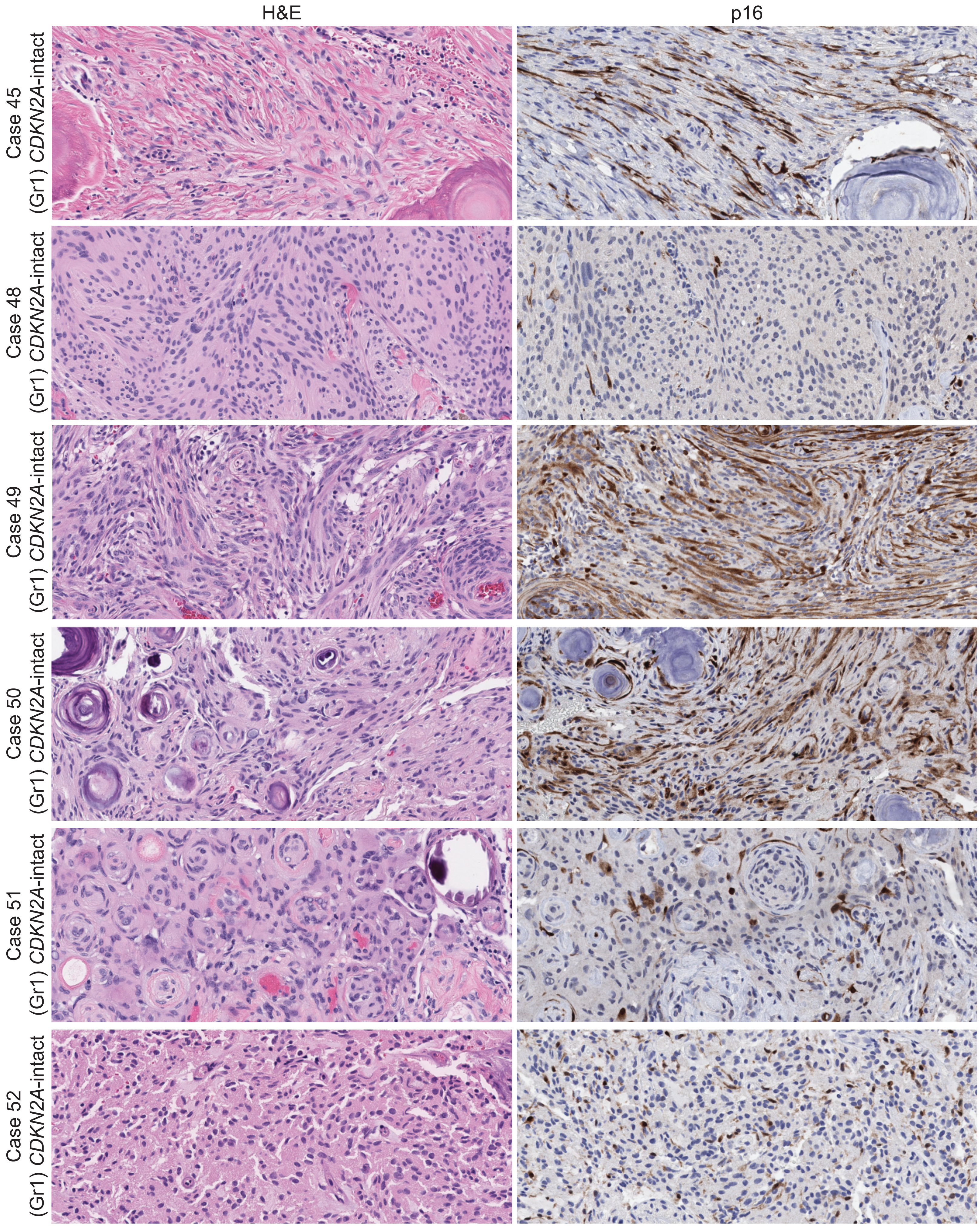

Supplementary Figure 2. Spectrum of p16 expression in CNS WHO grade 1 meningiomas with intact/wildtype *CDKN2A* alleles. 9/14 tumors (64%) demonstrated minimal to absent p16 immunoreactivity despite absence of *CDKN2A* alterations.
